# Statistical Model of Ocular Wavefronts With Accommodation

**DOI:** 10.1167/iovs.65.12.12

**Published:** 2024-10-08

**Authors:** María Mechó-García, María Arcas-Carbonell, Elvira Orduna-Hospital, Ana Sánchez-Cano, Norberto López-Gil, Rute J. Macedo-de-Araújo, Miguel Faria-Ribeiro, Paulo Fernandes, José Manuel González-Méijome, Jos Rozema

**Affiliations:** 1 Clinical and Experimental Optometry Research Lab, Physics Center of Minho and Porto Universities (CF-UM-UP), School of Sciences, University of Minho, Braga, Portugal; 2 Departamento de Física Aplicada, Universidad de Zaragoza, Zaragoza, España; 3 Aragon Health Research Institute (IIS Aragon), Zaragoza, Aragon, Spain; 4 Grupo de Ciencias de la Visión (CIVIUM), Universidad de Murcia, Murcia, Spain; 5 Visual Optics Lab Antwerp (VOLANTIS), Faculty of Medicine and Health Sciences, University of Antwerp, Antwerp, Belgium; 6 Department of Ophthalmology, Antwerp University Hospital, Edegem, Belgium

**Keywords:** statistical model, wavefront aberrations, zernike coefficients, accommodation

## Abstract

**Purpose:**

The purpose of this study was to determine the minimum number of orthonormal basis functions, applying Principal Component Analysis (PCA), to represent the most wavefront aberrations at different accommodation stages. The study also aims to generate synthetic wavefront data using these functions.

**Methods:**

Monocular wavefront data from 191 subjects (26.15 ± 5.56 years old) were measured with a Hartmann-Shack aberrometer, simulating accommodation from 0 diopters (D) to 5 D in 1 D steps. The wavefronts for each accommodative demand were rescaled for different pupil sizes: 4.66, 4.76, 4.40, 4.09, 4.07, and 3.68 mm. PCA was applied to 150 wavefront parameters (25 Zernike coefficients × 6 accommodation levels) to obtain eigenvectors for dimensional reduction. A total of 49 eigenvectors were modeled as a sum of 2 multivariate Gaussians, from which 1000 synthetic data sets were generated.

**Results:**

The first 49 eigenvectors preserved 99.97% of the original data variability. No significant differences were observed between the mean values and standard deviation of the generated and original 49 eigenvectors (two one-sided test [TOST], *P* > 0.05/49) and (F-test, *P* > 0.05/49), both with Bonferroni correction. The mean values of the generated parameters (1000) were statistically equal to those of the original data (TOST, *P* > 0.05/150). The variability of the generated data was similar to the original data for the most important Zernike coefficients (F-test, *P* > 0.05/150).

**Conclusions:**

PCA significantly reduces the dimensionality of wavefront aberration data across 6 accommodative demands, reducing the variable space by over 66%. The synthetic data generated by the proposed wavefront model for accommodation closely resemble the original clinical data.

Predicting visual performance and image quality through optical corrections for myopia and presbyopia across different accommodation levels is essential for optimizing subject outcomes. Accurate predictions enable the development of customized lenses and surgical techniques tailored to individual visual needs, improving clarity and comfort.^1^ For instance, contact lenses specifically designed for myopia control^2^ or presbyopia correction.^3^^,^^4^ Of particular importance is assessing the optical efficacy of bifocal and multifocal soft contact lenses intended for myopia correction in young patients with active accommodation.^5^^–^^8^ Evaluating their interaction with the natural focusing ability of young eyes versus presbyopic eyes is also crucial,^5^^,^^9^^,^^10^ as it ensures good optical quality under various accommodation conditions and provides valuable insights for improving optical devices.

Rigorous evaluation of these multifocal lenses involves large-scale clinical trials enrolling a significant number of participants. These trials are inherently time-consuming and resource-intensive, requiring considerable financial investment.^11^^,^^12^

For these reasons, eye models offer a valuable alternative, providing researchers with a simplified and readily available tool that mimics the human eye’s functionality.^13^ These models have been instrumental in the design of multifocal intraocular lenses (IOLs),^13^ the investigation of how optical aberrations impact retinal image quality,^14^^–^^16^ and the exploration of the eye’s visual performance across viewing distances following specific procedures.^5^

Scientific literature abounds with various schematic models of the human eye with different levels of complexity.^17^^–^^20^ Although some specific models like those proposed by Navarro et al.,^21^ Popiolek‐Masajada et al.,^22^^,^^23^ and Gullstrand,^17^ among others, offer utility for certain applications, they are often limited by their focus on a single accommodative state or by using average population data across multiple accommodative states.^21^ Once the human eye's morphological and physiological parameters exhibit significant individual variations around their average values, which creates a single, “ideal” eye model that captures the intricate details across a diverse population seems impractical. An alternative approach involves generative modeling, wherein synthetic wavefront datasets are generated with statistical properties mirroring those of the original data. Several such eye models have been proposed by Sorsby et al.,^24^ Thibos et al.,^25^ Zhao,^26^ Porter et al.,^27^ and Rozema et al.^28^^–^^31^ and allow, for example, analyzing the ocular biometry, intraocular lens performance, wavefront aberrations of well corrected and normal eyes, and keratoconus eyes. However, a crucial limitation remains – none of these models currently account for the eye’s ability to accommodate.

This study aims to investigate the de-correlation and dimensionality reduction of Zernike coefficients obtained under six accommodative states (0 diopters [D] to 5 D in 1 D increments) using Principal Component Analysis (PCA). The resulting reduced data is then used to fit a Gaussian multivariate model. This model is subsequently used to generate synthetic wavefront data with different accommodative demands. Finally, the generated synthetic data are compared to the original data for model validation.

## Methods

### Participants

This study analyzed monocular (right or left eyes, randomly selected) accommodative wavefront data from 191 healthy white European subjects (62 men and 129 women). Subjects were from three different institutions: the University of Minho, Braga (Portugal), the University of Zaragoza, Zaragoza (Spain), and the University of Murcia, Murcia (Spain). Their ages range from 18 to 40 years (mean ± standard deviation = 26.15 ± 5.56 years).

The average subjective refraction was –1.35 ± 2.22 D sphere and –0.53 ± 0.47 D cylinder. Exclusion criteria include ocular pathologies, accommodative problems (measured objectively), prior ocular surgery, refractive error outside the range of ±10 D sphere or exceeding 1 D cylinder, and a corrected visual acuity (VA) lower than 20/20 (measured with Early Treatment Diabetic Retinopathy Study [ETDRS] LogMAR chart).

The study adhered to the tenets of the Declaration of Helsinki and received approval from the Ethics Subcommittee for Life and Health Sciences of the University of Minho (Ref. 081/2022), the Clinical Research Ethics Committee of Aragón (CEICA; Ref. PI21-074), and the Ethics Committee of the University of Murcia. All participants signed informed consent forms after receiving a detailed explanation of the study’s purpose.

To maintain near-physiological conditions and natural accommodation, cycloplegics and mydriatics^32^^,^^33^ agents were not used.

### Wavefront Data

Wavefront aberrations from the second to the sixth order were measured at various target vergences using a commercial aberrometer (irx3; Imagine Eyes, Orsay, France). The irx3 incorporates a built-in fixation target, consisting of a black 6/12 Snellen letter “E” on a retro-illuminated white background. Subjects fixated on this target while an infrared beam (780 nm wavelength) projected from the device reached their retina. The aberrometer was programmed for automated measurements at accommodative demands ranging from 0 D to –5 D, in 1 D steps, using the internal Badal system.

Due to the inclusion of right eye (RE) and left eye (LE) in the dataset, Zernike coefficients with negative even meridional index and positive odd meridional index were sign-reversed for all LE data before analysis. This step accounts for the inherent mirror symmetries across the vertical meridian between RE and LE.

For accurate comparison, a fixed standard pupil diameter was used for each accommodation level 4.66 mm (0 D), 4.76 mm (1 D), 4.4 mm (2 D), 4.09 mm (3 D), 4.07 mm (4 D), and 3.68 mm (5 D). This standard size represents the largest pupil diameter achievable by 95% of the eyes under each accommodative state (fifth percentile), whereas the 5% of eyes with smaller pupil sizes were excluded for that particular vergence analysis to avoid extrapolation of the wavefront data. Eyes that were excluded for three or more vergences were excluded from the study altogether. Wavefront aberration measurements were then scaled down to this standard pupil diameter using the method described by Schwiegerling^34^ and further corrected by Visser et al.^35^

### Principal Components Analysis

As the data consist of 6 levels of accommodation, each characterized by 25 Zernike coefficients, there were originally 150 parameters to be included in the analysis. This number was sufficient for statistical modeling given the available sample size of 191 subjects.

**Figure 1.**
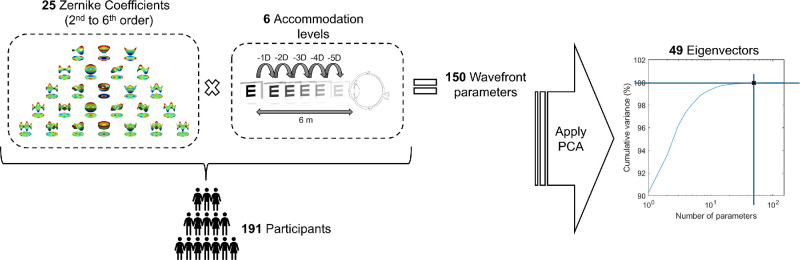
Diagram of the schematic process for obtaining the 49 eigenvectors. The intersection of the horizontal and vertical straight lines in the last graph indicates the point where 99.97% of the cumulative variance was found.

The database consisted of 150 parameters, representing the 6 levels of accommodation evaluated and 25 Zernike coefficients (from the second to the sixth orders) for each level. However, statistical modeling was performed on all 150 parameters, which was sufficiently large for the available sample size of 191 subjects. The schematic process can be seen in Figure 1.

Therefore, PCA^36^^,^^37^ a well-established dimensionality reduction technique, was used. PCA transforms the original Zernike coefficients into a new set of uncorrelated variables (eigenvectors) ordered by their contribution to the data’s variance. By choosing an appropriate cutoff, this method can be used for data reduction by selecting a subset of eigenvectors that captures most of the variability.

Here, a 99.97% variance cutoff was chosen across all accommodation levels to account for the large difference in amplitude between the defocus and spherical aberration terms. As illustrated in Figure 1 and Table 1 (see Supplementary Table S1 for details), this resulted in retaining 49 eigenvectors, which preserved 99.97% of the original data’s variability. The remaining 0.03%, primarily associated with higher-order aberrations with minimal practical impact on the model, was discarded. These retained eigenvectors formed an orthogonal and near-complete basis set for the data.

**Table 1.** Cumulative Variance (%) and Limits of the First 12 Eigenvectors

	% of Variance	Max, µm	Min, µm
EV 1	90.30	0.488	−0.011
EV 2	93.61	0.398	−0.294
EV 3	96.20	0.346	−0.445
EV 4	97.41	0.245	−0.472
EV 5	98.08	0.666	−0.424
EV 6	98.53	0.481	−0.483
EV 7	98.91	0.580	−0.571
EV 8	99.10	0.379	−0.242
EV 9	99.26	0.389	−0.643
EV 10	99.38	0.309	−0.324
EV 11	99.47	0.354	−0.235
EV 12	99.56	0.421	−0.295

### Gaussian Multivariate Model

The first 49 eigenvectors obtained by PCA were then used to construct the generative model. This model utilizes a linear combination of two multivariate Gaussian functions fitted using the Expectation-Maximization (EM) algorithm implemented in MATLAB 2022b (The MathWorks, Natick, MA, USA).^31^^,^^38^

This robust fitting procedure allows the model to generate a limitless number of random data points with a distribution that is statistically indistinguishable from that of the original data set.

In this study, 1000 such random data points were generated representing the coefficients of the 49 eigenvectors. These were subsequently transformed into Zernike coefficients, yielding a collection of 1000 synthetic wavefronts encompassing the 6 accommodative levels (0 D to 5 D in 1 D steps). Finally, these synthetic wavefronts can be directly compared with the original wavefront cohort.^39^

### Statistics

The statistical analysis aimed to establish equivalence between the original and synthetic datasets. Two one-sided tests (TOST)^40^^,^^41^ were used to define equivalence thresholds for the means of both sets. This approach ensures that any observed mean differences are likely due to chance, not systematic bias. Before TOST, the normality of the distribution was confirmed using Kolmogorov-Smirnov tests. Additionally, F-tests were conducted to compare the variability (variances) between original and generated data. All statistical tests were performed at a significance level of α = 0.05 and adjusted using Bonferroni correction for multiple simultaneous comparisons.

## Results

### Principal Components

**Figure 2.**
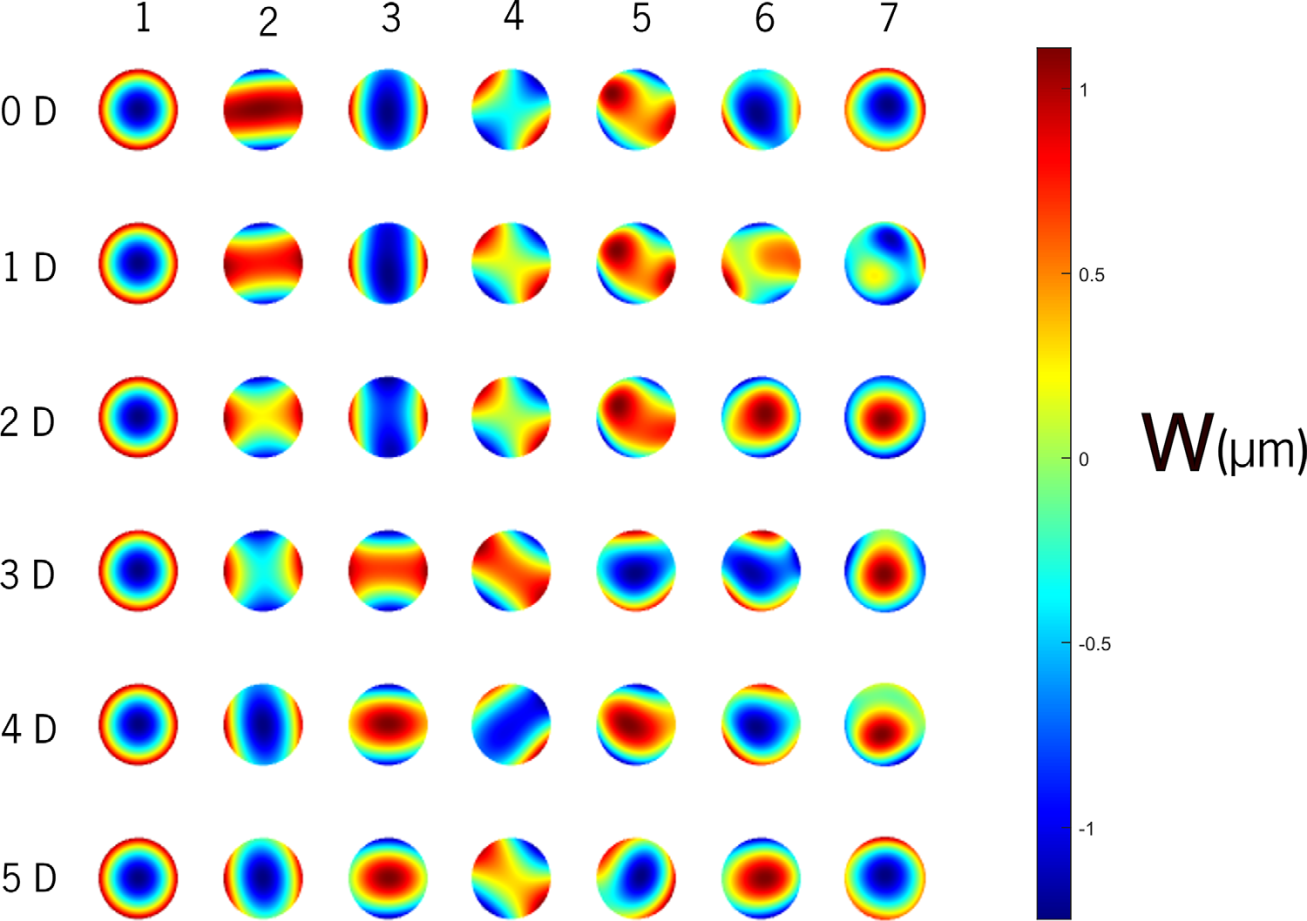
Base functions (eigenvectors) were obtained for the second to sixth order Zernike coefficients (*columns*) for six accommodative demands (*rows*). The wavefront of the first seven eigenvectors is ordered according to decreasing eigenvalues.

The resulting base functions derived from the initial wavefront data using PCA are shown in Figure 2 for the first 7 eigenvectors, each representing linear combinations of all 150 Zernike parameters across the 6 accommodative demands. Additional components up to the 49 eigenvectors are provided in Supplementary Figure S1. Only the first 49 orthonormal eigenvectors of the covariance matrix are considered, ordered by decreasing eigenvalues. Table 1 details the minimum and maximum limits for each eigenvector’s colormap (represented by blue and red in Fig. 2) and the cumulative variance explained by the first 12 components. The remaining details for components 13 to 49 can be found in Supplementary Table S1. The color maps (see Fig. 2, Supplementary Fig. S1) reveal that the first few eigenvectors, resemble rotated and distorted Zernike polynomials. For instance, eigenvector 1 appears similar to defocus, eigenvectors 2, 3, and 4 resemble astigmatism, eigenvectors 6 and 7 resemble spherical aberration, and eigenvector 8 resembles to coma. However, the patterns become more complex, exhibiting a higher-order mixing of Zernike coefficients with decreasing prominence of any single eigenvector.

The Zernike’s decomposition of the eigenvectors is represented by bar charts in Supplementary Figure S2, whereas Supplementary Table S2, from the supplements, gives a quantitative description of the base functions for the separate PCA by accommodative demands.

The first representing the most prominent component, is primarily composed of a C(2,0) Zernike term (parabolic revolution term). To analyze the following more complex eigenvectors, the indices *n* and *m* (*Z n,m*) were used as radial and angular frequencies, respectively. The one with the highest magnitude will be referred to as the fundamental eigenvector. The relative intensities percentage contribution to the root-mean-square (RMS) value of the eigenvector of the fundamental components are listed in Supplementary Table S2. Notably, the first 4 eigenvectors exhibit a dominant fundamental component exceeding 40% contribution. Conversely, eigenvectors from the fifth onward have relative intensities (< 40%) indicating a more complex composition with contributions from various Zernike terms. This implies that these later eigenvectors do not encode information solely from a single Zernike coefficient (please note that the relative contribution is given by the corresponding eigenvalue so that most of the variance is explained by the first eigenvectors).

### Validation

Statistical analysis confirmed that the distributions of all 49 eigenvectors in both the original and synthetic data sets were normally distributed (Kolmogorov-Smirnov test with Bonferroni correction, *P* > 0.001; Table 2, Supplementary Table S3). This indicates a good match between the statistical properties of the original data and the model's output. Furthermore, using the model to generate 1000 wavefronts across different accommodative demands and comparing the mean values of the eigenvectors with those of the original data, both are significantly equal (TOST with Bonferroni correction, *P* > 0.05/49; see Table 2, Supplementary Table S3). Finally, no significant differences were observed between the standard deviation of the 49 eigenvectors used to generate the model and the original data (F-test, with Bonferroni correction, *P* > 0.05/49; see Table 2, Supplementary Table S3).

**Table 2.** Comparison of the Eigenvectors of the Original Data (191) Versus the Generated Data (1000)

Parameter	KS^*^	Average (SD) Original Data, µm	Average (SD) Generated Data, µm	TOST	F-Test^*^
EV 1	0.075	0.000 (3.907)	−0.283 (3.692)	0.337	0.294
EV 2	0.330	0.000 (0.749)	0.004 (0.696)	0.946	0.176
EV 3	0.165	0.000 (0.661)	−0.005 (0.651)	0.922	0.765
EV 4	0.008	0.000 (0.453)	0.029 (0.450)	0.418	0.868
EV 5	0.000	0.000 (0.335)	0.003 (0.336)	0.916	0.999
EV 6	0.061	0.000 (0.276)	0.004 (0.284)	0.860	0.636
EV 7	0.541	0.000 (0.255)	−0.007 (0.250)	0.715	0.722
EV 8	0.668	0.000 (0.179)	0.003 (0.176)	0.826	0.764
EV 9	0.454	0.000 (0.164)	0.003 (0.162)	0.816	0.750
EV 10	0.382	0.000 (0.141)	−0.004 (0.137)	0.709	0.546
EV 11	0.485	0.000 (0.125)	0.001 (0.125)	0.905	0.982
EV 12	0.593	0.000 (0.121)	<0.001 (0.126)	0.990	0.467

KS, Kolmogorov-Smirnov test for normality; SD, standard deviation.

* *P* < 0.05/49 = 4.17·10-3 (Bonferroni correction) indicates a significant difference.

**Figure 3.**
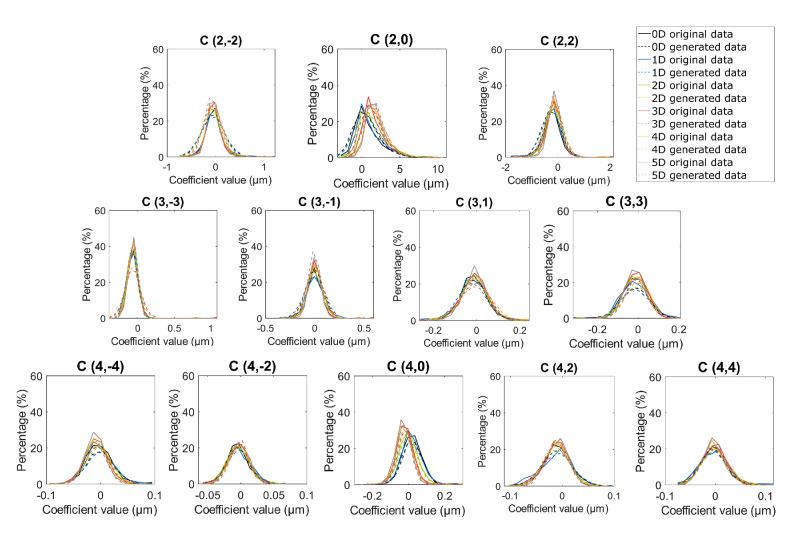
Distribution of Zernike coefficients up to the fourth order across subjects, illustrating the percentage of subjects exhibiting each coefficient value for each Zernike mode, for each accommodative state, 0 D (*black*), 1 D (*blue*), 2 D (*green*), 3 D (*red*), 4 D (*orange*), and 5 D (*brown*), for the original data (191 eyes, *solid line*) and generated data (1000 eyes, *dashed line*).

The distribution of Zernike coefficients up to the sixth order in each accommodative target vergence of the generated data is well-aligned with that of the original data, as depicted in Figure 3 and Supplementary Figure S3.

The 150 parameters (Zernike coefficients) are normally distributed within both the original 191 eyes and the 1000 synthetic eyes (Kolmogorov-Smirnov test with Bonferroni correction, *P* > 0.05/150; Table 3, Supplementary Table S4). The wavefronts generated for each of the accommodative vergences are significantly equal to the mean values of the original data (TOST, *P* > 0.05/150; see Table 3, Supplementary Table S4). Although no statistically significant differences were observed between the standard deviations of the original data and generated data for coefficients up to fourth order (F-test, *P* > 0.05/150; see Table 3, Supplementary Table S4), some of the high-order Zernike coefficients (beyond the fifth order) showed statistically significant differences, mainly for higher accommodative demands (F-test, *P* < 0.05/150; see Table 3, Supplementary Table S4). The agreement with the original data versus the generated data can also be seen in the distributions presented in Figure 3 up to the fourth order. The figure shows the calculated Zernike coefficients up to the sixth order for 1000 generated wavefronts (dashed lines, color-coded for each accommodative demand) compared to the measured coefficients from the original data (solid lines), Supplementary Figure S3 provides details on the contents of the fifth and sixth order Zernike coefficients.

**Table 3.** Comparison of the Zernike Coefficients for Each Accommodative Demand From the Original Data (191) Versus the Generated Data (1000)

Parameters		KS	Average (SD) Original Data, µM	Average (SD) Generated Data, µM	TOST	F-Test
C (2,-2)	0 D	0.034	0.017 (0.213)	0.011 (0.213)	0.547	0.818
	1 D	0.051	0.021 (0.226)	0.010 (0.227)	0.341	0.797
	2 D	0.009	0.015 (0.192)	0.006 (0.194)	0.877	0.846
	3 D	0.089	0.014 (0.157)	0.002 (0.156)	0.933	0.722
	4 D	0.069	0.007 (0.158)	−0.003 (0.158)	0.915	0.709
	5 D	0.019	0.005 (0.135)	−0.005 (0.134)	0.954	0.649
C (2,0)	0 D	0.095	1.249 (1.747)	1.143 (1.674)	0.341	0.327
	1 D	0.056	1.544 (1.902)	1.418 (1.818)	0.877	0.282
	2 D	0.064	1.826 (1.757)	1.736 (1.669)	0.933	0.258
	3 D	0.173	1.948 (1.390)	1.890 (1.367)	0.915	0.500
	4 D	0.220	2.335 (1.493)	2.284 (1.415)	0.954	0.259
	5 D	0.146	2.278 (1.312)	2.227 (1.235)	0.849	0.231
C (2,2)	0 D	0.427	−0.159 (0.362)	−0.153 (0.331)	0.877	0.455
	1 D	0.298	−0.129 (0.366)	−0.120 (0.342)	0.868	0.481
	2 D	0.409	−0.090 (0.318)	−0.083 (0.294)	0.835	0.452
	3 D	0.128	−0.066 (0.271)	−0.046 (0.309)	0.995	0.885
	4 D	0.119	−0.035 (0.271)	−0.030 (0.293)	0.964	0.768
	5 D	0.561	−0.020 (0.227)	−0.017 (0.229)	0.796	0.822
C (3,-1)	0 D	0.674	0.024 (0.082)	0.024 (0.081)	0.915	0.792
	1 D	0.803	0.026 (0.091)	0.024 (0.093)	0.872	0.806
	2 D	0.848	0.026 (0.074)	0.026 (0.076)	0.909	0.886
	3 D	0.158	0.023 (0.063)	0.025 (0.076)	0.887	0.578
	4 D	0.297	0.025 (0.067)	0.029 (0.071)	0.981	0.594
	5 D	0.453	0.022 (0.052)	0.024 (0.052)	0.985	0.796
C (3,1)	0 D	0.465	<0.001 (0.060)	0.002 (0.059)	0.954	0.721
	1 D	0.803	−0.005 (0.067)	−0.002 (0.065)	0.906	0.656
	2 D	0.694	0.002 (0.059)	0.004 (0.057)	0.740	0.712
	3 D	0.554	0.007 (0.057)	0.008 (0.060)	0.887	0.980
	4 D	0.573	0.011 (0.066)	0.012 (0.067)	0.960	0.892
	5 D	0.267	0.011 (0.060)	0.011 (0.061)	0.863	0.647
C (4,0)	0 D	0.718	0.039 (0.050)	0.036 (0.050)	0.407	0.549
	1 D	0.753	0.036 (0.056)	0.033 (0.054)	0.437	0.661
	2 D	0.382	0.016 (0.049)	0.014 (0.049)	0.550	0.746
	3 D	0.808	0.002 (0.041)	<0.001 (0.040)	0.660	0.499
	4 D	0.968	−0.004 (0.048)	−0.007 (0.046)	0.614	0.492
	5 D	0.858	−0.008 (0.041)	−0.010 (0.038)	0.880	0.300
C (6,0)	0 D	0.831	−0.001 (0.013)	−0.002 (0.010)	0.580	0.010
	1 D	0.166	−0.001 (0.014)	−0.002 (0.011)	0.419	0.001
	2 D	0.112	−0.001 (0.013)	−0.002 (0.010)	0.480	**<0.001** ^*^
	3 D	0.231	−0.001 (0.009)	−0.002 (0.007)	0.651	0.036
	4 D	0.134	−0.002 (0.009)	−0.002 (0.008)	0.766	0.016
	5 D	0.749	−0.001 (0.006)	−0.001 (0.005)	0.883	0.002

F-test, compare variances; KS, Kolmogorov-Smirnov test for normality; SD, standard deviation; TOST, two one-sided tests.

* *P* < 0.05/150 = 3.33·10-4 (Bonferroni correction) indicates a significant difference.

## Discussion

The statistical wavefront model with accommodation successfully allowed to generate an unlimited number of synthetic wavefronts encompassing six different accommodative demands. These synthetic wavefronts exhibit statistically indistinguishable from the original data used for model development (see Tables 2, 3), falling within the established tolerance levels. Notably, the model effectively preserves the variability of key accommodative Zernike coefficients, such as defocus and primary spherical aberration coefficients, closely resembling those observed in the original data (see Fig. 3).

This study introduces a novel compact and accurate generative model for accommodative wavefront errors in a large young adult population aged 18 to 40 years. The model stands out by realistically representing intersubject variability through a series of Zernike coefficients, reflecting the dynamic nature of accommodation, without the biological variability presented before.^17^^,^^18^^,^^20^^,^^21^^,^^42^^–^^44^ By using an EM algorithm,^31^^,^^38^ it became possible to effectively fit the multivariate distribution of the 49 eigenvectors, representing 99.97% of the population variance. This provides researchers in physiological optics and simulation design with a powerful tool. The generated wavefronts offer a multitude of applications, including simulating retinal image quality, determining tolerances for accommodation-related wavefront errors, designing multifocal contact lenses that account for accommodation-induced changes in optical quality, and guiding wavefront-based contact lenses design. They can also facilitate virtual clinical trials for multifocal contact lenses and the evaluation of optical quality in subjects with different accommodative states using various multifocal contact lens designs.

Similar to our study, Porter et al.^27^ applied PCA to analyze wavefront aberrations. They tried to reduce the dimensions of 18 Zernike coefficients across 109 subjects and identified 6 eigenvectors. The first of the eigenvectors captured 90% of the variance, primarily contained defocus information, which is consistent with our findings. Spherical aberration information was primarily captured in the sixth eigenvector in the Porter et al.^27^ study, as in our study, the more significant information related to spherical aberration is found in the sixth and seventh eigenvector.

Although directly using all Zernike coefficients for modeling is possible, the high number of parameters involved can lead to instability. Using PCA instead offers a powerful alternative because it has the advantage of parameter reduction based on desired accuracy.^29^^,^^36^^,^^37^ In this study, 49 eigenvectors capture explain 99.97% of the data variability of the data. This enables constructing a model with varying complexity and accuracy, ranging from a basic mean-only model precisely tailored to the specific application. Additionally, PCA eliminates existing correlations among Zernike coefficients, establishing a comprehensive and fully independent framework for the multivariate distribution. The resulting eigenvectors’ coefficients are statistically independent, allowing for improved analysis. Finally, eigenvectors can be physically interpreted as combinations of aberration terms contributing to the population’s increased variability. As shown in Supplementary Figure S1, the first eigenvectors closely resemble Zernike polynomials, albeit increasingly distorted as the expansion progresses. It is important to center the data (subtract the average) before applying PCA to avoid introducing bias. The average value (bias) is then added back after generating random data to obtain the final wavefront values, see Table 2.

However, the model has several inherent limitations to consider. First, the model derives its features from the original data, which means that it cannot improve the information content beyond that initial data set. Second, eigenvector compression reduces the variability, which affects the higher-order wavefront aberrations (F-test in Supplementary Table S4). However, reassuringly, Table 3 demonstrates that the variability of key accommodation-dependent Zernike coefficients^45^^–^^53^ exhibit variability comparable to that observed in the original data (see Table 3). This suggests that the reduced higher-order variability likely has minimal impact on the model's performance. Third, the model accounts for the decrease in pupil size with accommodative demand and calculates wavefront accordingly. However, it uses a fixed pupil size for each level of accommodative demand. This means the pupil size at the maximum 5 D accommodation demand is always set to 3.68 mm, which may not perfectly reflect reality. Finally, due to the measurement method the study is limited to monochromatic aberrations. Therefore, the results cannot be directly extrapolated to aberrations at different wavelengths.

The previously used Thibos et al.^25^ wavefront model addressed this challenge by using a stochastic algorithm designed to generate a wide range of synthetic wavefronts from a database of 200 eyes, although valuable for many years, the model had notable limitations including that it did not account for the inherent correlation between wavefronts from both eyes, which could introduce bias. These concerns were addressed in the models proposed by Rozema et al.^28^^,^^30^^,^^31^ which involved including only one of the eyes per individual, eliminating the bias from the inter-eye correlations. Additionally, they use a Bigaussian model and eyes with uncorrected ametropia, overcoming further shortcomings identified in previous studies.

The wide variability in wavefront characteristics observed in the general population, especially when accommodation is involved, cannot be accurately captured by a fixed set of parameters, typical of classical eye models. Whereas useful for first-order approximations for optical calculations in eyes with average dimensions, these models may yield less realistic results for eyes deviating from this average. Introducing a stochastic model addresses this limitation by considering the entire population wavefront data rather than solely relying on the population mean. This approach allows to generate a vast number of synthetic wavefronts, forming a diverse cohort that closely mirrors the original dataset. These synthetic eyes hold the potential to become a valuable alternative for researchers lacking access to real wavefront data, particularly for evaluating optical quality metrics in myopia control treatments.

## Conclusions

This study introduces a novel statistical wavefront model that incorporates accommodation, overcoming the limitations of traditional fixed-parameter models. The model effectively captures the wavefront variability associated with accommodation, enabling the generation of a vast number of synthetic wavefronts with realistic accommodative states. These synthetic wavefronts closely resemble the original data and hold immense value for researchers in several ways such as: performing optometry calculations with greater accuracy; testing, and evaluating the impact of various optical devices on retinal image quality across different accommodation stages; and facilitating research for those lacking access to real wavefront data.

## Supplementary Material

Supplement 1
