# Supplementary material for: Statistical Model of Ocular Wavefronts With Accommodation
Source: Invest Ophthalmol Vis Sci. 2024 Oct 8;65(12):12. doi: 10.1167/iovs.65.12.12 (PMC11463707; doi:10.1167/iovs.65.12.12)
Supplement: Supplement 1 [file iovs-65-12-12_s001.pdf]

Supplement

TABLE S1: CUMULATIVE VARIANCE (%) AND LIMITS FOR THE REMAINING 49

	% of variance	max (µm)	min(µm)
EV 13	99.63	0.349	-0.207
EV 14	99.68	0.204	-0.355
EV 15	99.72	0.499	-0.529
EV 16	99.75	0.476	-0.518
EV 17	99.77	0.241	-0.338
EV 18	99.79	0.288	-0.246
EV 19	99.81	0.468	-0.498
EV 20	99.82	0.582	-0.251
EV 21	99.84	0.372	-0.342
EV 22	99.85	0.324	-0.370
EV 23	99.86	0.358	-0.304
EV 24	99.87	0.276	-0.359
EV 25	99.88	0.295	-0.221
EV 26	99.88	0.219	-0.435
EV 27	99.89	0.350	-0.303
EV 28	99.90	0.382	-0.325
EV 29	99.90	0.334	-0.267
EV 30	99.91	0.359	-0.337
EV 31	99.91	0.237	-0.435
EV 32	99.92	0.244	-0.304
EV 33	99.92	0.255	-0.263
EV 34	99.93	0.236	-0.260
EV 35	99.93	0.319	-0.373
EV 36	99.94	0.266	-0.214
EV 37	99.94	0.354	-0.261
EV 38	99.94	0.244	-0.245
EV 39	99.94	0.217	-0.340
EV 40	99.95	0.238	-0.281
EV 41	99.95	0.268	-0.249
EV 42	99.95	0.227	-0.293
EV 43	99.96	0.277	-0.243
EV 44	99.99	0.240	-0.332
EV 45	99.96	0.297	-0.280
EV 46	99.96	0.248	-0.250
EV 47	99.96	0.381	-0.246
EV 48	99.96	0.321	-0.230
EV 49	99.97	0.278	-0.294

Supplement

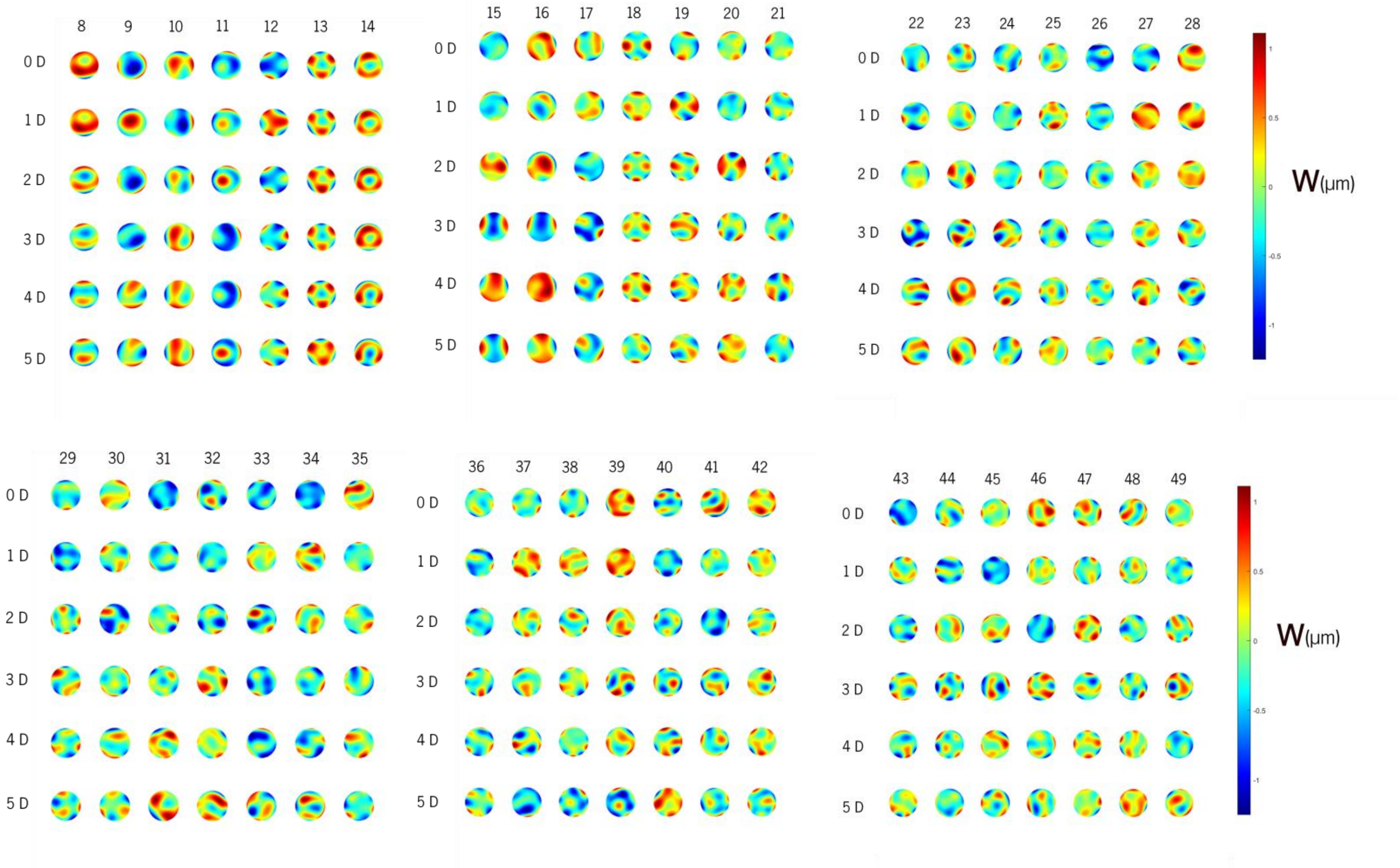

Figure S1. Base functions (eigenvectors) obtained for the 2nd to 6th order Zernike coefficients (columns) for six accommodative demands (rows). The wavefront from the 8th to the 49th eigenvectors ordered according to decreasing eigenvalues.

Supplement

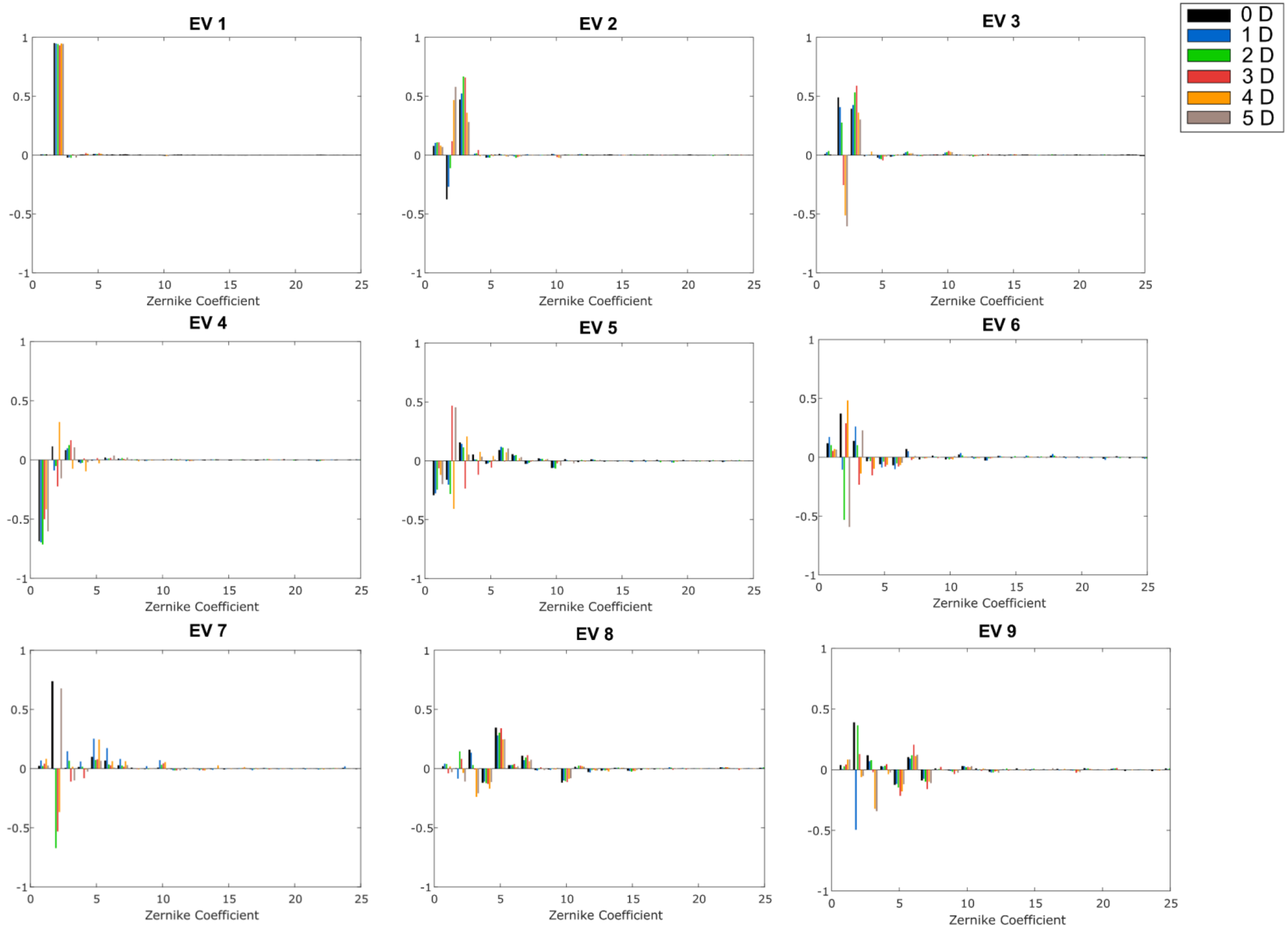

EV 1
EV 2
EV 3
EV 4
EV 5
EV 6
EV 7
EV 8
EV 9
Zernike Coefficient
0 D
1 D
2 D
3 D
4 D
5 D

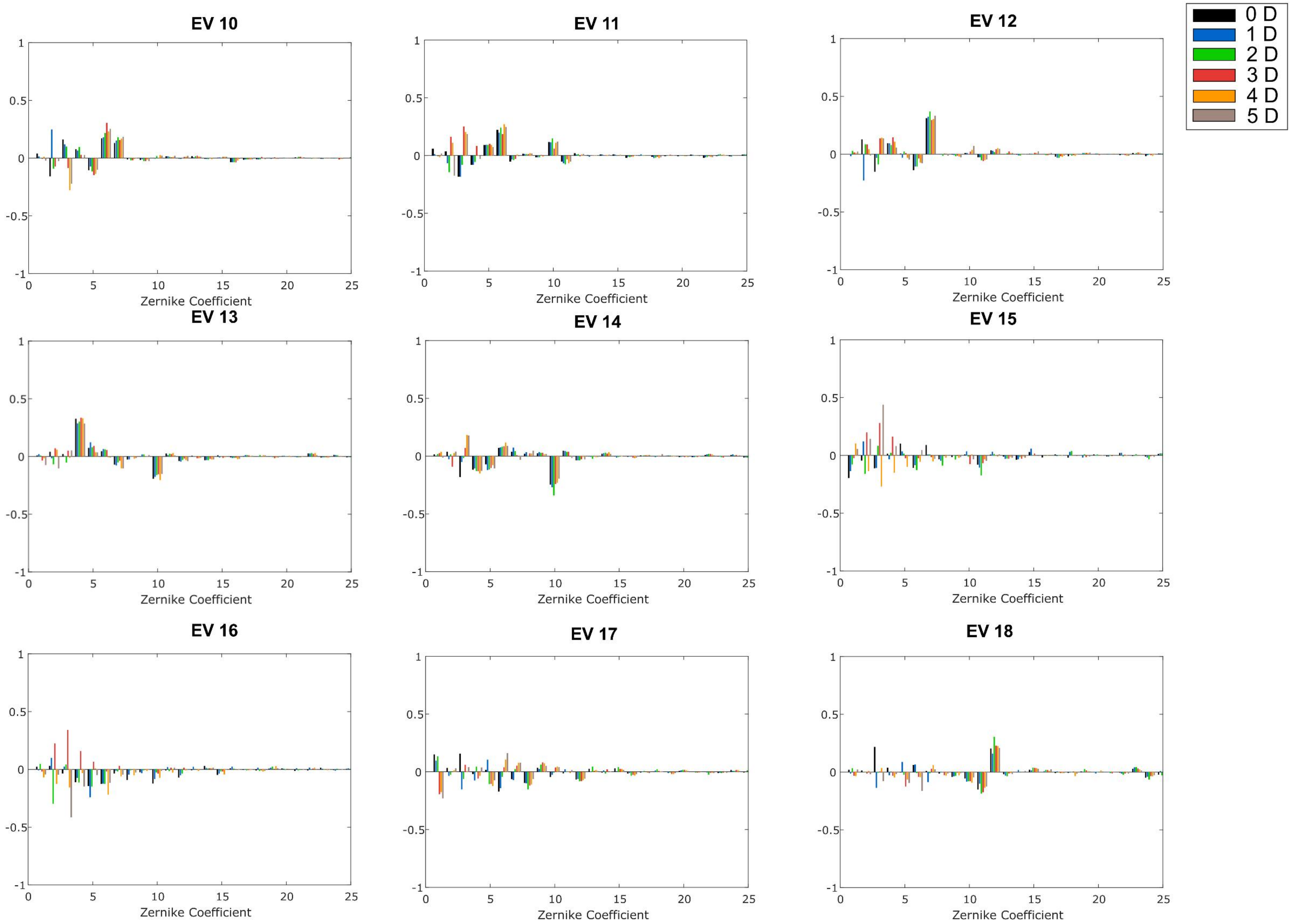
EV 10
EV 11
EV 12
EV 13
EV 14
EV 15
EV 16
EV 17
EV 18
Zernike Coefficient
0 D
1 D
2 D
3 D
4 D
5 D

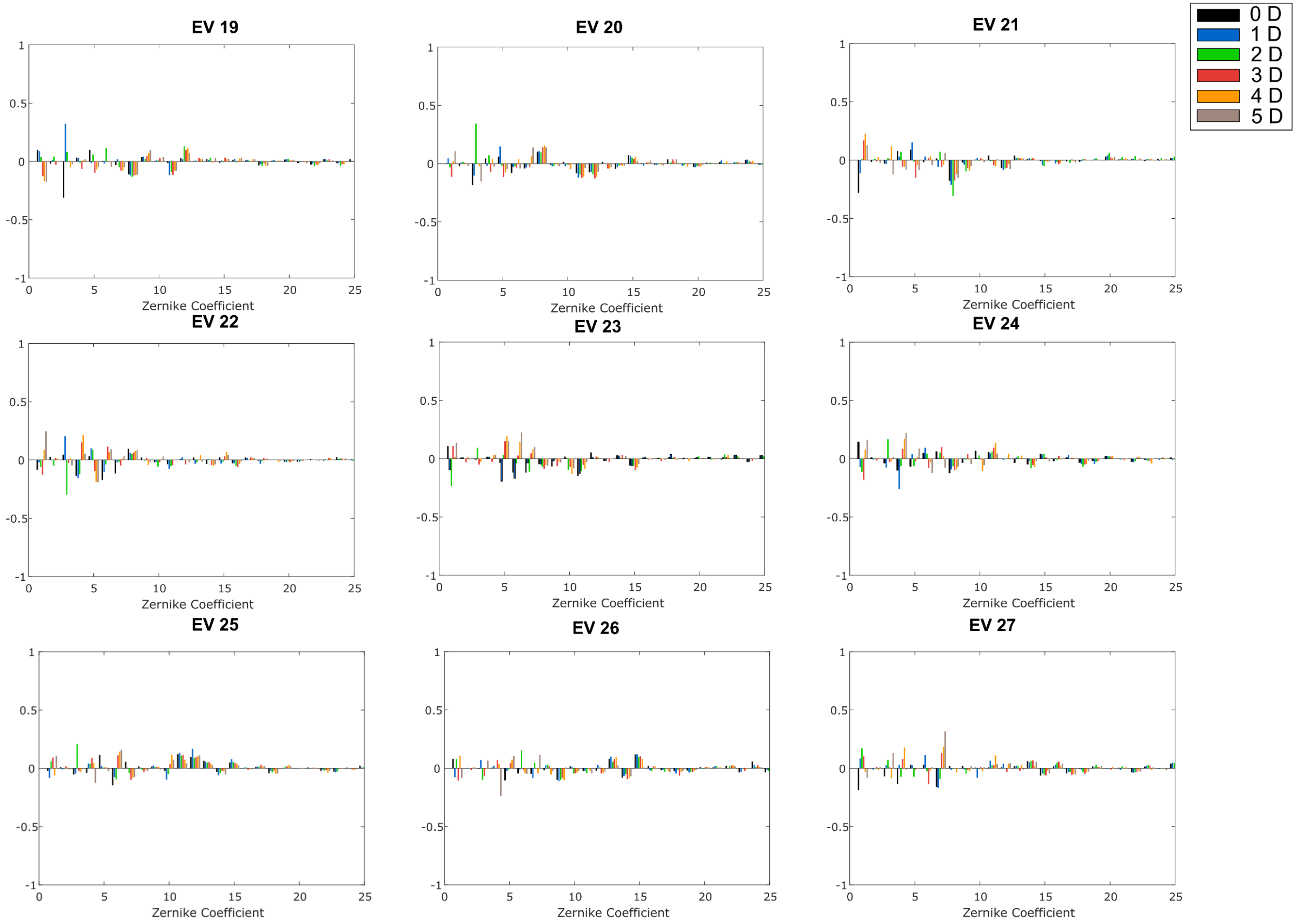

EV 19
EV 20
EV 21
EV 22
EV 23
EV 24
EV 25
EV 26
EV 27
Zernike Coefficient
0 D
1 D
2 D
3 D
4 D
5 D

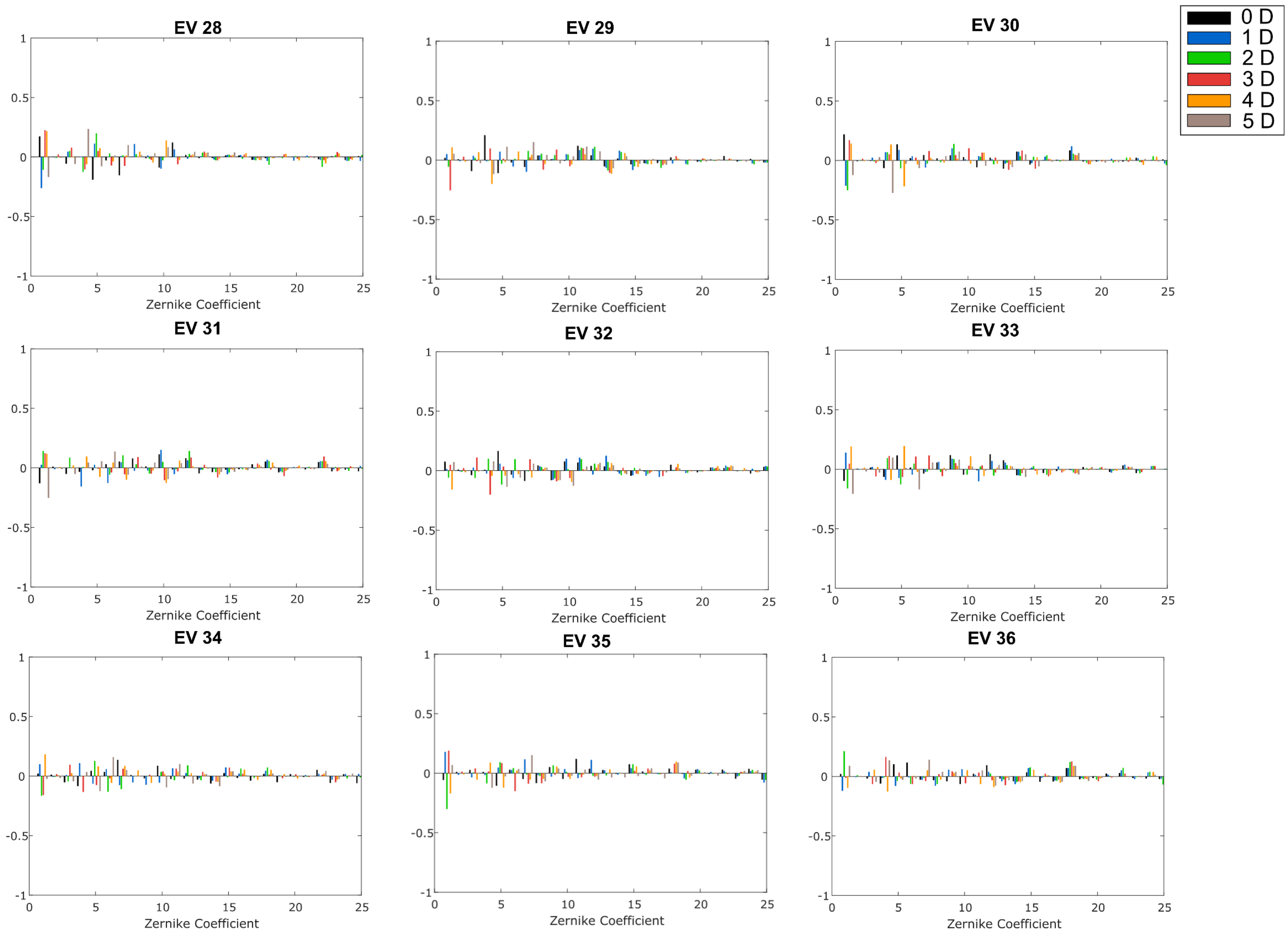
EV 28
EV 29
EV 30
EV 31
EV 32
EV 33
EV 34
EV 35
EV 36
Zernike Coefficient
0 D
1 D
2 D
3 D
4 D
5 D

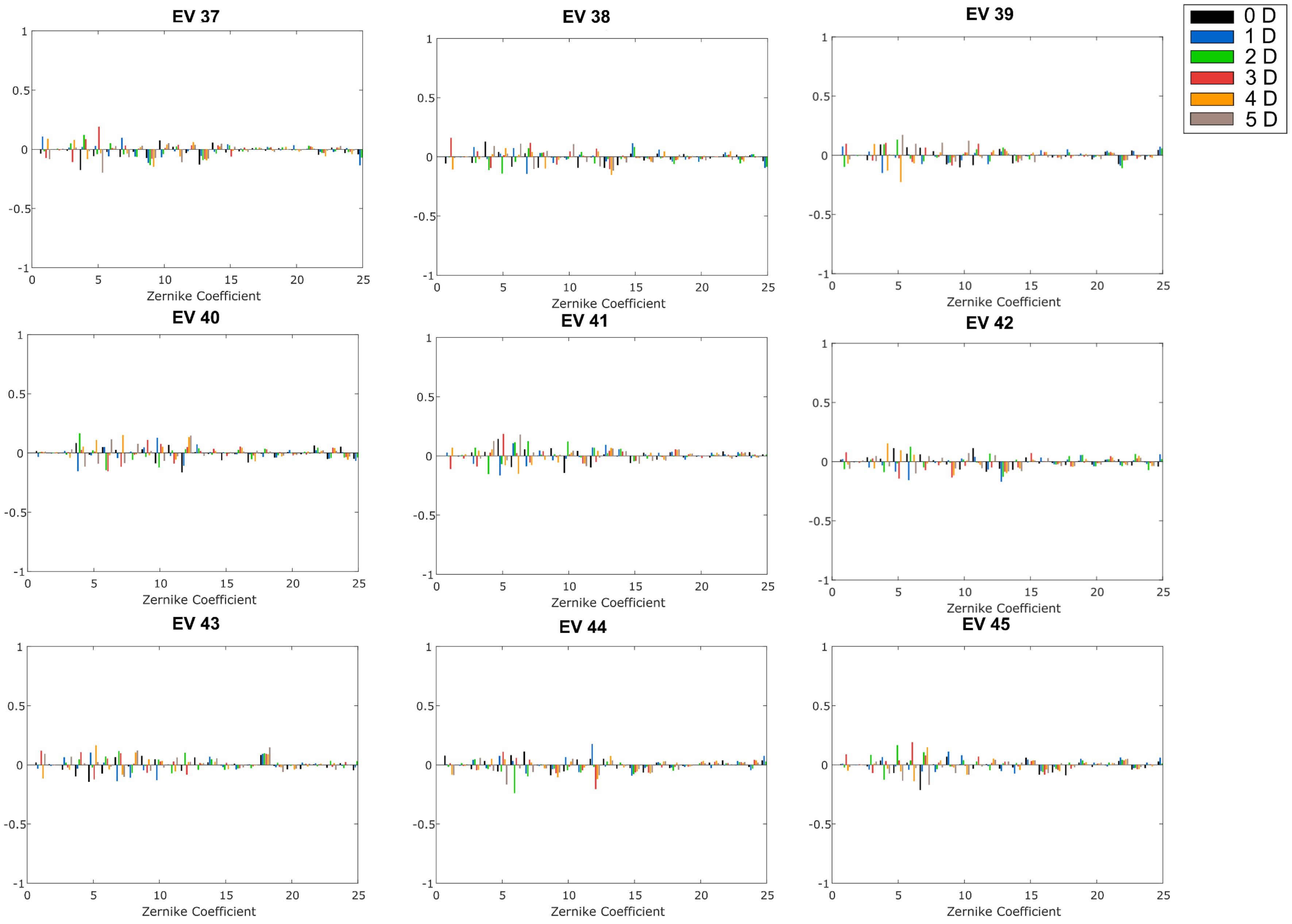

EV 37
EV 38
EV 39
EV 40
EV 41
EV 42
EV 43
EV 44
EV 45
Zernike Coefficient
0 D
1 D
2 D
3 D
4 D
5 D

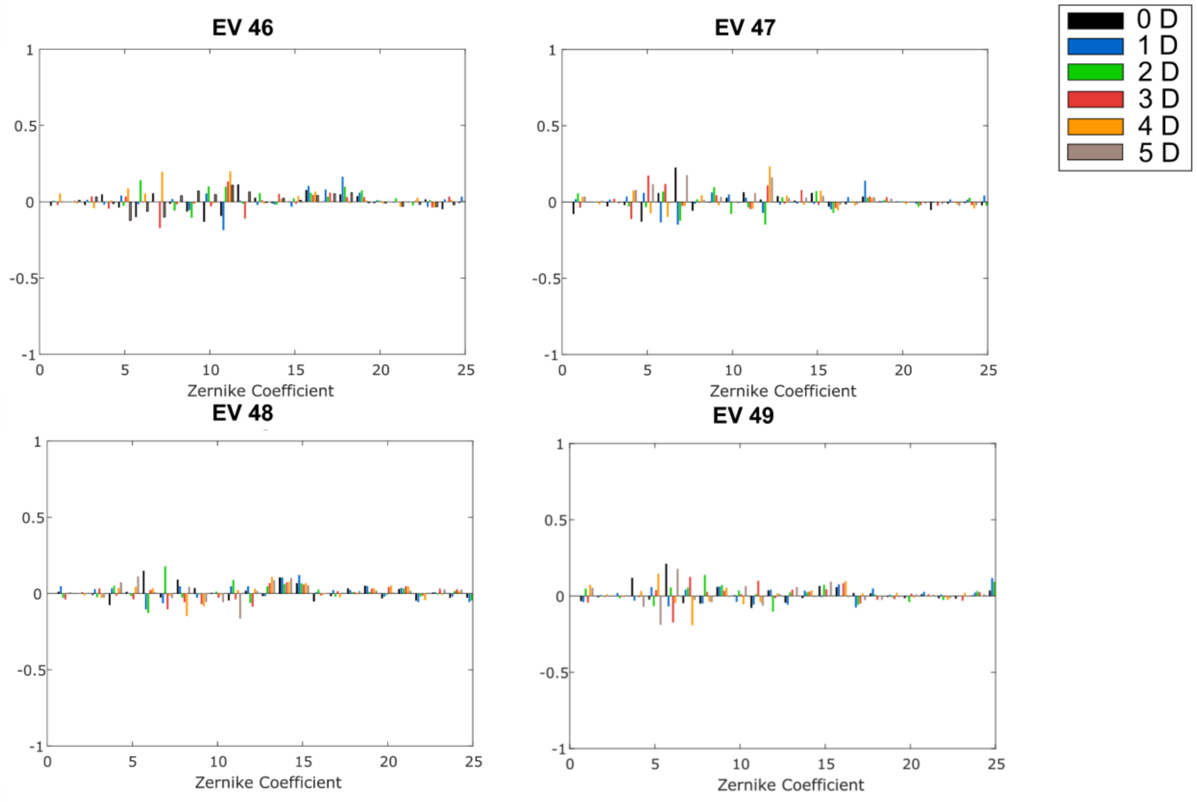

Figure S2. Bar diagrams showing the distribution of Zernike coefficients for the first 49 eigenvectors (EV) corresponding to Supplement 2. Zernike coefficients for the unaccommodated eye (black), accommodative demand of 1 D (blue), 2 D (green), 3 D (red), 4 D (orange) and 5 D (brown).

Supplement

TABLE S2: FUNDAMENTAL COMPONENTS FOR EACH ACCOMMODATIVE DEMAND OF EACH EIGENVECTOR, AND PERCENTAGE CONTRIBUTION TO THE MODULUS OF THE EIGENVECTOR.

	0 D		1 D		2 D		3 D		4 D		5 D	
**EV**	**FUNDAMENTAL**	**% OF RMS**	**FUNDAMENTAL**	**% OF RMS**	**FUNDAMENTAL**	**% OF RMS**	**FUNDAMENTAL**	**% OF RMS**	**FUNDAMENTAL**	**% OF RMS**	**FUNDAMENTAL**	**% OF RMS**
1	C_2^0	95.06	C_2^0	94.77	C_2^0	94.39	C_2^0	93.18	C_2^0	94.88	C_2^0	94.52
2	C_2^2	47.21	C_2^2	52.43	C_2^2	66.74	C_2^2	65.89	C_2^0	46.76	C_2^0	57.96
3	C_2^0	48.95	C_2^2	42.63	C_2^2	53.20	C_2^2	59.03	C_2^0	51.28	C_2^0	60.53
4	C_2^{-2}	68.60	C_2^{-2}	69.56	C_2^{-2}	71.21	C_2^{-2}	50.12	C_2^{-2}	41.64	C_2^{-2}	60.14
5	C_2^{-2}	29.06	C_2^{-2}	27.56	C_2^0	28.10	C_2^0	46.88	C_2^0	40.91	C_2^0	45.47
6	C_2^0	36.85	C_2^2	26.04	C_2^0	53.24	C_2^0	28.79	C_2^0	48.24	C_2^0	59.30
7	C_2^0	73.58	C_3^{-1}	25.26	C_2^0	67.18	C_2^0	53.09	C_2^0	36.76	C_2^0	67.74
8	C_3^{-1}	34.38	C_3^{-1}	27.92	C_3^{-1}	30.23	C_3^{-1}	33.99	C_3^{-1}	24.55	C_3^{-1}	24.82
9	C_2^0	38.69	C_2^0	49.56	C_2^0	36.55	C_3^{-1}	21.65	C_2^2	32.19	C_2^2	34.09
10	C_3^1	17.18	C_2^0	24.76	C_3^1	21.72	C_3^1	30.45	C_3^1	23.15	C_3^1	25.25
11	C_3^1	21.98	C_2^2	18.32	C_3^1	24.06	C_2^2	25.28	C_3^1	27.20	C_3^1	24.87
12	C_3^3	31.12	C_3^3	32.47	C_3^3	37.07	C_3^3	29.56	C_3^3	30.28	C_3^3	33.29
13	C_3^{-3}	32.66	C_3^{-3}	28.60	C_3^{-3}	30.24	C_3^{-3}	33.54	C_3^{-3}	33.07	C_3^{-3}	28.51
14	C_4^0	24.61	C_4^0	26.99	C_4^0	34.02	C_4^0	24.22	C_4^0	23.13	C_4^0	19.50
15	C_2^{-2}	19.50	C_2^{-2}	13.66	C_2^0	16.04	C_2^2	28.14	C_2^2	26.95	C_2^2	43.96
16	C_3^{-1}	14.56	C_3^{-1}	24.06	C_2^0	29.70	C_2^2	34.13	C_3^1	21.92	C_2^2	41.68
17	C_3^1	17.12	C_2^2	15.26	C_2^{-2}	13.40	C_2^{-2}	19.43	C_2^{-2}	17.59	C_2^{-2}	22.87
18	C_2^2	21.76	C_4^4	15.88	C_4^4	30.54	C_4^4	22.41	C_4^4	22.41	C_4^4	20.85
19	C_2^2	31.01	C_2^2	32.28	C_4^4	13.01	C_2^{-2}	12.67	C_2^{-2}	16.91	C_2^{-2}	17.66
20	C_2^2	18.52	C_3^{-1}	14.69	C_2^2	34.32	C_4^{-4}	13.89	C_4^{-4}	15.59	C_2^2	15.32
21	C_2^{-2}	28.18	C_4^{-4}	21.04	C_4^{-4}	30.60	C_4^{-4}	17.57	C_2^{-2}	22.60	C_4^{-4}	15.35
22	C_3^1	17.11	C_2^2	20.30	C_2^2	29.83	C_3^{-3}	15.03	C_3^{-3}	21.40	C_2^{-2}	24.64
23	C_4^2	14.62	C_3^{-1}	19.40	C_2^{-2}	23.40	C_3^{-1}	14.87	C_3^{-1}	19.40	C_3^1	22.43
24	C_2^{-2}	14.41	C_3^{-3}	25.90	C_2^2	16.73	C_2^{-2}	18.07	C_3^{-3}	17.14	C_3^{-3}	21.98
25	C_3^1	14.70	C_4^4	16.68	C_2^2	20.80	C_4^2	11.31	C_3^1	14.33	C_3^1	15.76
26	C_5^{-1}	11.80	C_5^{-1}	12.05	C_3^1	15.32	C_2^{-2}	10.68	C_2^{-2}	10.63	C_3^{-3}	23.67

TABLE S2: FUNDAMENTAL COMPONENTS FOR EACH ACCOMMODATIVE DEMAND OF EACH EIGENVECTOR, AND PERCENTAGE CONTRIBUTION TO THE MODULUS OF THE EIGENVECTOR.

	**0 D**		**1 D**		**2 D**		**3 D**		**4 D**		**5 D**	
**EV**	**FUNDAMENTAL**	**% OF RMS**	**FUNDAMENTAL**	**% OF RMS**	**FUNDAMENTAL**	**% OF RMS**	**FUNDAMENTAL**	**% OF RMS**	**FUNDAMENTAL**	**% OF RMS**	**FUNDAMENTAL**	**% OF RMS**
27	C_2^{-2}	18.82	C_3^3	16.75	C_2^{-2}	17.17	C_3^1	13.77	C_3^3	18.35	C_3^3	31.62
28	C_3^{-1}	19.03	C_2^{-2}	26.21	C_3^{-1}	19.94	C_2^{-2}	22.54	C_2^{-2}	21.67	C_3^{-3}	23.61
29	C_3^{-3}	21.00	C_3^3	9.78	C_4^4	11.25	C_2^{-2}	25.42	C_3^{-3}	19.98	C_3^3	15.19
30	C_2^{-2}	21.92	C_2^{-2}	21.40	C_2^{-2}	25.18	C_2^{-2}	17.01	C_3^{-1}	21.68	C_3^{-3}	27.29
31	C_2^{-2}	12.95	C_3^{-3}	15.67	C_4^4	14.25	C_2^{-2}	12.44	C_4^0	12.74	C_2^{-2}	25.17
32	C_3^{-1}	16.55	C_5^{-5}	12.58	C_3^{-1}	11.60	C_3^{-3}	20.11	C_2^{-2}	15.98	C_3^{-1}	13.61
33	C_4^4	12.57	C_2^{-2}	13.85	C_2^{-2}	16.08	C_3^3	11.53	C_3^{-1}	19.50	C_2^{-2}	20.65
34	C_3^3	13.59	C_3^{-3}	10.87	C_2^{-2}	16.57	C_2^{-2}	16.02	C_2^{-2}	18.37	C_3^1	15.90
35	C_4^2	12.05	C_2^{-2}	17.93	C_2^{-2}	30.06	C_2^{-2}	18.96	C_2^{-2}	16.93	C_3^3	15.18
36	C_3^1	11.64	C_2^{-2}	12.21	C_2^{-2}	21.19	C_3^{-3}	16.43	C_3^{-3}	12.73	C_3^3	14.09
37	C_3^{-3}	17.38	C_4^{-2}	11.53	C_4^{-2}	13.48	C_3^{-1}	18.99	C_4^{-2}	14.70	C_3^{-1}	19.71
38	C_3^{-3}	12.63	C_3^3	14.34	C_3^{-1}	14.18	C_2^{-2}	16.04	C_5^{-5}	15.21	C_5^{-5}	11.66
39	C_4^0	10.23	C_3^{-3}	15.00	C_3^{-1}	13.24	C_3^{-3}	10.44	C_3^{-1}	22.63	C_3^{-1}	17.25
40	C_4^4	16.38	C_3^{-3}	15.34	C_3^{-3}	16.74	C_3^1	15.43	C_3^3	15.18	C_4^4	14.67
41	C_4^0	14.34	C_3^{-1}	16.51	C_3^{-3}	15.40	C_3^{-1}	18.58	C_3^1	15.11	C_3^1	17.95
42	C_4^2	11.42	C_3^1	15.68	C_5^{-5}	12.81	C_3^{-1}	14.36	C_3^{-3}	15.41	C_3^1	9.96
43	C_3^{-1}	14.35	C_3^3	13.75	C_3^3	11.56	C_3^{-1}	12.25	C_3^{-1}	16.54	C_5^5	14.78
44	C_3^3	11.33	C_4^4	17.65	C_3^1	23.89	C_4^4	20.56	C_4^4	12.15	C_3^{-1}	16.55
45	C_3^3	21.22	C_4^{-2}	11.25	C_3^{-1}	16.58	C_3^1	19.03	C_3^3	14.97	C_3^3	16.92
46	C_4^0	13.05	C_4^2	18.59	C_3^3	17.23	C_4^2	19.86	C_3^3	19.74	C_3^{-1}	12.24
47	C_3^3	22.58	C_3^3	14.81	C_4^4	14.66	C_3^{-1}	17.39	C_4^4	23.36	C_3^3	17.71
48	C_3^1	14.93	C_5^{-1}	12.11	C_3^3	17.73	C_3^3	10.33	C_4^{-4}	14.80	C_4^2	16.46
49	C_3^1	21.15	C_6^6	11.77	C_4^{-4}	13.80	C_3^1	17.36	C_3^3	19.15	C_3^{-1}	18.84

Supplement

TABLE S3: COMPARISON OF THE REMAINING 49 EIGENVECTORS OF THE ORIGINAL DATA (191) VS THE GENERATED DATA (1,000)

Parameter	KS*	Average (SD) Original data (µm)	Average (SD) Generated data (µm)	TOST	F-test*
EV 13	0.966	0.000 (0.112)	0.004 (0.109)	0.624	0.715
EV 14	0.766	0.000 (0.088)	0.004 (0.088)	0.533	0.982
EV 15	0.052	0.000 (0.086)	-0.005 (0.086)	0.499	0.942
EV 16	0.443	0.000 (0.077)	-0.002 (0.077)	0.707	0.996
EV 17	0.341	0.000 (0.059)	0.003 (0.060)	0.496	0.791
EV 18	0.265	0.000 (0.055)	0.001 (0.056)	0.742	0.918
EV 19	0.161	0.000 (0.054)	0.002 (0.055)	0.607	0.701
EV 20	0.599	0.000 (0.049)	0.000 (0.048)	0.936	0.839
EV 21	0.601	0.000 (0.046)	-0.001 (0.047)	0.708	0.849
EV 22	0.639	0.000 (0.044)	-0.002 (0.044)	0.579	0.946
EV 23	0.196	0.000 (0.042)	0.001 (0.041)	0.751	0.838
EV 24	0.882	0.000 (0.041)	0.000 (0.040)	0.927	0.922
EV 25	0.717	0.000 (0.038)	-0.001 (0.038)	0.848	0.926
EV 26	0.250	0.000 (0.035)	0.002 (0.034)	0.449	0.545
EV 27	0.221	0.000 (0.036)	0.000 (0.033)	0.927	0.744
EV 28	0.852	0.000 (0.033)	0.000 (0.033)	0.936	0.837
EV 29	0.943	0.000 (0.032)	-0.001 (0.032)	0.802	0.981
EV 30	0.770	0.000 (0.030)	0.000 (0.030)	0.935	0.676
EV 31	0.545	0.000 (0.029)	0.000 (0.030)	0.905	0.807
EV 32	0.830	0.000 (0.029)	0.000 (0.028)	0.833	0.684
EV 33	0.862	0.000 (0.028)	0.000 (0.028)	0.999	0.923
EV 34	0.700	0.000 (0.027)	0.001 (0.027)	0.733	0.906
EV 35	0.613	0.000 (0.026)	-0.001 (0.026)	0.661	0.655
EV 36	0.339	0.000 (0.026)	-0.001 (0.026)	0.716	0.939
EV 37	0.465	0.000 (0.024)	0.000 (0.024)	0.988	0.792
EV 38	0.306	0.000 (0.023)	-0.001 (0.023)	0.607	0.821
EV 39	0.479	0.000 (0.023)	0.001 (0.023)	0.629	0.988
EV 40	0.910	0.000 (0.022)	0.001 (0.022)	0.754	0.748
EV 41	0.934	0.000 (0.022)	0.000 (0.022)	0.797	0.692
EV 42	0.464	0.000 (0.021)	0.000 (0.021)	0.859	0.495
EV 43	0.254	0.000 (0.021)	0.000 (0.021)	0.845	0.901
EV 44	0.937	0.000 (0.020)	0.001 (0.020)	0.751	0.785
EV 45	0.968	0.000 (0.020)	-0.001 (0.020)	0.632	0.765
EV 46	0.357	0.000 (0.019)	0.001 (0.019)	0.483	0.900
EV 47	0.988	0.000 (0.019)	0.000 (0.018)	0.839	0.687
EV 48	0.916	0.000 (0.018)	0.000 (0.018)	0.968	0.877
EV 49	0.667	0.000 (0.018)	0.000 (0.017)	0.684	0.601

SD: Standard deviation

KS: Kolmogorov-Smirnov test for normality

* p-value < 0.05/49 = 4.17·10-3 (Bonferroni correction) indicates a significant difference **(in bold).**

Supplement

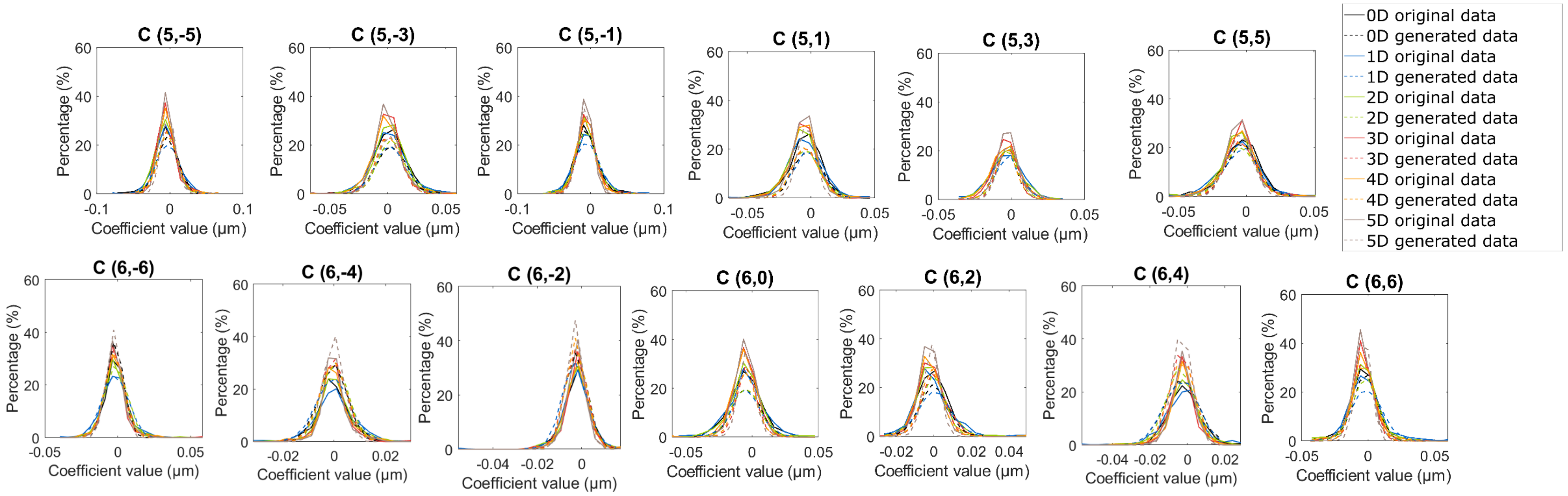

Figure S3. Zernike coefficients distribution of 5th and 6th order across subjects, illustrating the percentage of subjects exhibiting each coefficient value for each Zernike mode, for each accommodative state, 0 D (black), 1 D (blue), 2 D (green), 3 D (red), 4 D (orange) and 5 D (brown), for the original data (191 eyes, solid line) and generated data (1,000 eyes, dashed line).

Supplement

TABLE S4: COMPARISON OF THE ZERNIKE COEFFICIENTS FOR EACH ACCOMMODATIVE DEMAND FROM THE ORIGINAL DATA (191) VS THE GENERATED DATA (1,000).

Parameters		KS	Average (SD) original data (μm)	Average (SD) generated data	TOST	T-test
**C (3,-3)**	0 D	0.349	-0.039 (0.058)	-0.039 (0.056)	0.933	0.493
	1 D	0.885	-0.043 (0.061)	-0.043 (0.058)	0.829	0.416
	2 D	0.928	-0.040 (0.055)	-0.039 (0.054)	0.946	0.601
	3 D	<0.001	-0.036 (0.046)	-0.033 (0.097)	0.668	0.900
	4 D	0.001	-0.036 (0.053)	-0.035 (0.083)	0.702	0.920
	5 D	0.500	-0.025 (0.046)	-0.026 (0.051)	0.837	0.558
**C (3,3)**	0 D	0.604	0.002 (0.060)	0.002 (0.063)	0.849	0.432
	1 D	0.820	0.003 (0.068)	0.001 (0.070)	0.743	0.812
	2 D	0.576	0.005 (0.060)	0.003 (0.061)	0.734	0.948
	3 D	0.787	0.005 (0.048)	0.005 (0.049)	0.786	0.859
	4 D	0.993	0.009 (0.054)	0.009 (0.055)	0.840	0.969
	5 D	0.967	0.007 (0.051)	0.006 (0.049)	0.897	0.442
**C (4,-4)**	0 D	0.924	0.005 (0.025)	0.005 (0.023)	0.768	0.079
	1 D	0.537	0.006 (0.027)	0.005 (0.025)	0.727	0.087
	2 D	0.654	0.001 (0.023)	0.001 (0.020)	0.829	0.061
	3 D	0.958	-0.001 (0.021)	-0.001 (0.020)	0.755	0.214
	4 D	0.747	-0.001 (0.021)	-0.001 (0.019)	0.597	0.047
	5 D	0.436	-0.003 (0.020)	-0.004 (0.017)	0.840	0.005
**C (4,-2)**	0 D	0.890	-0.001 (0.017)	-0.001 (0.015)	0.937	0.016
	1 D	0.442	0.002 (0.019)	0.001 (0.017)	0.877	0.041
	2 D	0.841	<0.001 (0.017)	<0.001 (0.015)	0.962	0.011
	3 D	0.761	0.001 (0.016)	0.001 (0.014)	0.854	0.079
	4 D	0.541	0.001 (0.016)	0.002 (0.015)	0.717	0.052
	5 D	0.657	0.002 (0.014)	0.003 (0.013)	0.430	0.026
**C (4,2)**	0 D	0.933	-0.003 (0.026)	-0.003 (0.025)	0.843	0.511
	1 D	0.335	-0.005 (0.029)	-0.005 (0.029)	0.764	0.512
	2 D	0.837	-0.005 (0.025)	-0.005 (0.024)	0.998	0.525
	3 D	0.947	-0.006 (0.023)	-0.005 (0.022)	0.911	0.278
	4 D	0.803	-0.006 (0.019)	-0.005 (0.023)	0.982	0.396
	5 D	0.644	-0.003 (0.019)	-0.003 (0.017)	0.700	0.047
**C (4,4)**	0 D	0.810	0.004 (0.026)	0.003 (0.024)	0.849	0.161
	1 D	0.815	0.005 (0.030)	0.004 (0.028)	0.667	0.248
	2 D	0.930	0.006 (0.026)	0.006 (0.025)	0.895	0.447
	3 D	0.443	0.005 (0.023)	0.005 (0.022)	0.737	0.483
	4 D	0.399	0.005 (0.024)	0.005 (0.024)	0.997	0.814
	5 D	0.803	0.005 (0.023)	0.004 (0.021)	0.845	0.179
**C (5,-5)**	0 D	0.581	<-0.001 (0.016)	<0.001 (0.014)	0.700	0.023
	1 D	0.214	0.001 (0.017)	0.001 (0.015)	0.718	0.158
	2 D	0.671	-0.001 (0.013)	<0.001 (0.012)	0.713	0.127
	3 D	0.018	0.001 (0.013)	0.001 (0.012)	0.585	0.052
	4 D	0.087	0.001 (0.013)	0.001 (0.012)	0.708	0.041
	5 D	0.029	0.002 (0.011)	0.002 (0.009)	0.539	**<0.001***
**C (5,-3)**	0 D	0.956	0.006 (0.014)	0.006 (0.013)	0.991	0.051
	1 D	0.980	0.006 (0.015)	0.006 (0.013)	0.698	0.004
	2 D	0.592	0.006 (0.013)	0.006 (0.011)	0.982	0.026
	3 D	0.235	0.004 (0.012)	0.004 (0.010)	0.787	0.004
	4 D	0.531	0.004 (0.012)	0.004 (0.010)	0.917	0.002
	5 D	0.178	0.004 (0.012)	0.005 (0.011)	0.893	0.029
**C (5,-1)**	0 D	0.824	-0.001 (0.016)	-0.001 (0.014)	0.888	0.069
	1 D	0.253	-0.001 (0.019)	<0.001 (0.018)	0.913	0.122
	2 D	0.266	-0.001 (0.015)	-0.001 (0.014)	0.813	0.119
	3 D	0.193	-0.001 (0.012)	<0.001 (0.011)	0.847	0.035
	4 D	0.370	-0.002 (0.013)	-0.002 (0.011)	0.952	0.065
	5 D	0.441	-0.002 (0.011)	-0.002 (0.008)	0.851	**<0.001***
**C (5,1)**	0 D	0.432	-0.001 (0.013)	<0.001 (0.010)	0.364	0.001
	1 D	0.583	-0.001 (0.014)	<0.001 (0.011)	0.333	**<0.001***
	2 D	0.736	-0.002 (0.013)	-0.002 (0.010)	0.365	0.002
	3 D	0.285	-0.002 (0.011)	-0.001 (0.010)	0.439	0.109
	4 D	0.327	-0.002 (0.011)	-0.002 (0.010)	0.386	0.007
	5 D	0.903	-0.002 (0.009)	-0.001 (0.007)	0.824	**<0.001***
**C (5,3)**	0 D	0.293	-0.003 (0.010)	-0.002 (0.007)	0.959	0.001
	1 D	0.198	-0.001 (0.011)	-0.001 (0.009)	0.839	**<0.001***
	2 D	0.114	-0.001 (0.010)	-0.001 (0.008)	0.671	**<0.001***

TABLE S4: COMPARISON OF THE ZERNIKE COEFFICIENTS FOR EACH ACCOMMODATIVE DEMAND FROM THE ORIGINAL DATA (191) VS THE GENERATED DATA (1,000).

Parameters		KS	Average (SD) original data (μm)	Average (SD) generated data	TOST	T-test
	3 D	0.520	-0.001 (0.008)	-0.001 (0.006)	0.864	**<0.001***
	4 D	0.242	-0.002 (0.009)	-0.002 (0.007)	0.950	0.001
	5 D	0.972	-0.001 (0.007)	<0.001 (0.006)	0.857	**<0.001***
**C (5,5)**	0 D	0.841	-0.001 (0.013)	<0.001 (0.011)	0.735	**<0.001***
	1 D	0.384	-0.001 (0.015)	-0.001 (0.012)	0.653	**<0.001***
	2 D	0.954	-0.002 (0.014)	-0.002 (0.011)	0.614	**<0.001***
	3 D	0.208	-0.001 (0.012)	-0.001 (0.010)	0.761	0.025
	4 D	0.747	-0.001 (0.012)	-0.002 (0.010)	0.805	0.010
	5 D	0.556	-0.002 (0.011)	-0.002 (0.008)	0.933	**<0.001***
**C (6,-6)**	0 D	0.100	0.001 (0.007)	0.001 (0.005)	0.935	**<0.001***
	1 D	0.740	0.001 (0.010)	0.001 (0.008)	0.772	**<0.001***
	2 D	0.031	0.001 (0.009)	0.001 (0.007)	0.937	**<0.001***
	3 D	0.625	0.001 (0.008)	<0.001 (0.006)	0.880	**<0.001***
	4 D	0.628	0.001 (0.007)	0.001 (0.006)	0.884	**<0.001***
	5 D	0.468	0.001 (0.006)	<0.001 (0.004)	0.736	**<0.001***
**C (6,-4)**	0 D	0.500	0.002 (0.007)	0.002 (0.005)	0.957	**<0.001***
	1 D	0.605	0.002 (0.008)	0.002 (0.006)	0.947	**<0.001***
	2 D	0.378	0.002 (0.006)	0.002 (0.005)	0.899	**<0.001***
	3 D	0.181	0.001 (0.005)	0.002 (0.004)	0.789	**<0.001***
	4 D	0.034	0.002 (0.006)	0.002 (0.005)	0.966	**<0.001***
	5 D	0.015	0.002 (0.005)	0.002 (0.003)	0.915	**<0.001***
**C (6,-2)**	0 D	0.270	<-0.001 (0.006)	<0.001 (0.004)	0.991	**<0.001***
	1 D	0.841	-0.001 (0.007)	-0.001 (0.005)	0.614	**<0.001***
	2 D	0.773	-0.001 (0.006)	<0.001(0.004)	0.978	**<0.001***
	3 D	0.174	-0.001 (0.005)	<0.001 (0.004)	0.943	**<0.001***
	4 D	0.133	-0.001 (0.005)	<0.001 (0.003)	0.672	**<0.001***
	5 D	0.310	-0.001 (0.004)	-0.001 (0.003)	0.640	**<0.001***
**C (6,2)**	0 D	0.884	0.002 (0.008)	0.002 (0.007)	0.885	0.028
	1 D	0.010	0.002 (0.010)	0.002 (0.008)	0.686	0.001
	2 D	0.087	0.002 (0.008)	0.002 (0.006)	0.767	**<0.001***
	3 D	0.037	0.002 (0.007)	0.002 (0.006)	0.692	0.008
	4 D	0.120	0.001 (0.007)	0.001 (0.006)	0.930	**<0.001***
	5 D	0.052	0.001 (0.005)	0.001 (0.003)	0.824	**<0.001***
**C (6,4)**	0 D	0.540	-0.001 (0.010)	-0.001 (0.008)	0.888	0.007
	1 D	0.972	-0.001 (0.010)	-0.001 (0.008)	0.902	**<0.001***
	2 D	0.339	-0.001 (0.008)	-0.001 (0.007)	0.666	0.001
	3 D	0.396	-0.001 (0.006)	<0.001 (0.005)	0.690	**<0.001***
	4 D	0.303	-0.001 (0.008)	<0.001 (0.006)	0.964	0.001
	5 D	0.169	-0.001 (0.006)	<0.001 (0.004)	0.817	**<0.001***
**C (6,6)**	0 D	0.989	0.001 (0.010)	<0.001 (0.008)	0.995	**<0.001***
	1 D	0.016	0.001 (0.013)	0.002 (0.011)	0.657	0.008
	2 D	0.129	-0.001 (0.011)	<0.001 (0.009)	0.721	0.001
	3 D	0.192	-0.001 (0.008)	-0.001 (0.006)	0.936	**<0.001***
	4 D	0.440	0.001 (0.009)	<0.001 (0.007)	0.799	**<0.001***
	5 D	0.409	-0.001 (0.006)	<0.001 (0.004)	0.841	**<0.001***

SD: Standard deviation.

KS: Kolmogorov-Smirnov Test for Normality.

TOST: Two One-Sided Tests.

F-test: Compare variances.

* p-value < 0.05/150 = 3.33·10-4 (Bonferroni correction) indicates a significant difference **(in bold).**
